# Postoperative visual loss following cerebral arteriovenous malformation surgery: a case report

**DOI:** 10.12688/f1000research.3-27.v2

**Published:** 2014-03-14

**Authors:** Nicolai Goettel, Jayati Ghosh, Michael Tymianski, Pirjo H Manninen

**Affiliations:** 1 Department of Anesthesia, University Health Network, Toronto Western Hospital, Toronto, ON, Canada; 2 Department of Anesthesia and Intensive Care Medicine, University Hospital Basel, Basel, Switzerland; 3 Division of Neurosurgery, University Health Network, Toronto Western Hospital, Toronto, ON, Canada

## Abstract

We report the case of a 46 year-old woman presenting with unilateral postoperative visual loss after right frontal craniotomy for resection of an arteriovenous malformation in the supine position.

The intraoperative course was uneventful with maintenance of hemodynamic stability. Blood loss was 300 ml; postoperative hemoglobin was 12.4 g/dl. In the recovery room, the patient reported loss of vision in her right eye. Ophthalmologic examination revealed decreased visual acuity, color vision, and visual field. Assessment of the retina was normal, but the patient showed a relative afferent pupillary defect consistent with the clinical diagnosis of ischemic optic neuropathy. Postoperative computer tomogram showed normal perfusion of ophthalmic artery and vein, no hemorrhage or signs of cerebral ischemia or edema. The patient recovered most of her vision 3 months after surgery.

Anesthesiologists should be aware that this condition may follow uncomplicated intracranial surgeries in the supine position, and should obtain prompt ophthalmologic consultation when a patient develops postoperative visual loss.

## Introduction

Postoperative visual loss (POVL) is a known complication of surgery and anesthesia; its incidence varies from 0.03% after spine surgery to 0.086% after cardiac surgery ^1^. Typically, this involves ischemic optic neuropathy (ION), and is clinically characterized by the acute or subacute loss of visual acuity and/or visual field. Both anterior (diffuse optic disc swelling) and posterior (no optic disc swelling) ION have been reported after general anesthesia in spinal and other non-ocular surgeries ^1– 3^. The incidence of POVL in a general surgical population is low (0.0012%) ^1^; to our knowledge, only one case has previously been reported in intracranial neurovascular surgery, and was included in the POVL registry of the American Society of Anesthesiologists (ASA) ^4^.

## Case report

**Figure 1.**
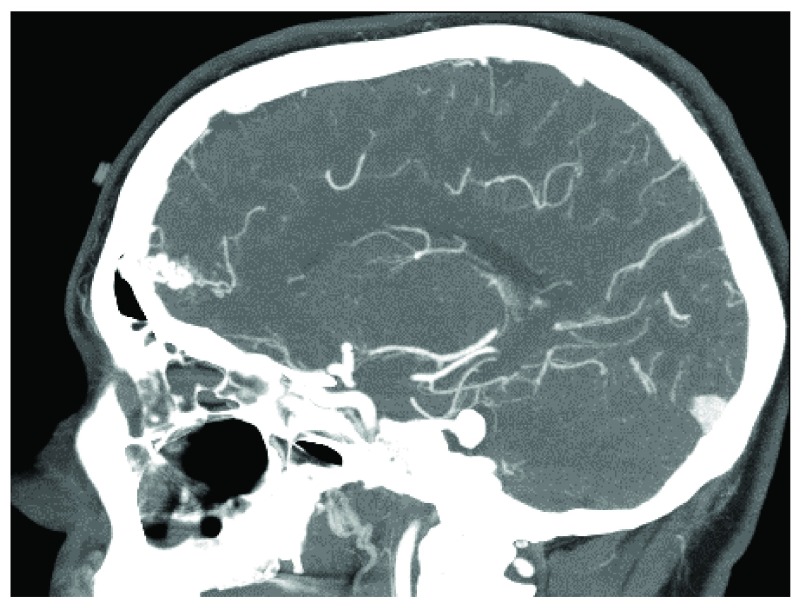
Preoperative computerized tomographic angiography showing the frontal AVM (Spetzler-Martin grade I).

A 46 year-old woman initially presented with paresthesia in hands and legs, and was diagnosed with a right frontal, subcortical arteriovenous malformation (AVM) of 1.5 cm in diameter. The AVM was classified as grade I according to Spetzler-Martin criteria ^5^ (small size, non-eloquence of adjacent brain, and only superficial venous drainage). Preoperative cerebral angiography showed that arterial blood supply of the AVM was via a frontal cortical branch and a superior frontopolar branch originating from the anterior communicating artery. Venous drainage of the AVM was superficial. Embolization prior to the craniotomy was not performed. Figure 1 shows the preoperative computer tomography (CT) angiogram.

Patient weight was 91 kg and height 165 cm (body mass index 33). The medical history revealed arterial hypertension, as well as coronary artery disease with previous myocardial infarction (MI) and percutaneous coronary angioplasty. Preoperative transthoracic echocardiography showed a grade II left ventricular function (ejection fraction 50–55%) and mild distal anteroseptal and distal anterior wall hypokinesia. The patient ceased to smoke one year ago with a 30 pack-year history. Apart from distal paresthesia in the extremities, the patient’s neurological status was normal. There was no history of impaired vision or previous eye surgery. Her medications consisted of metoprolol 12.5 mg twice daily, aspirin 81 mg and clopidogrel 75 mg once daily. Aspirin and clopidogrel were stopped 7 days prior to the intervention. Preoperative blood pressure was 130/90 mmHg, and hemoglobin concentration was 14.2 g/dl.

The patient was classified as ASA physical status 3, and underwent craniotomy with surgical excision of the AVM. After induction of general anesthesia with midazolam 2 mg, fentanyl 150 μg, and propofol 150 mg, followed by neuromuscular blockade with rocuronium 50 mg, the patient’s trachea was orally intubated. Anesthesia was maintained with sevoflurane and a remifentanil infusion during the 4-hour surgical procedure. Surgery was performed in the supine position, with the head elevated to 30 degrees and fixation in a head frame. The patient’s intraoperative blood pressure was maintained stable around 100 mmHg systolic, and there was minimal blood loss (300 ml). Total crystalloid infusion was 2 l. Emergence from anesthesia was uneventful, and the patient was extubated while awake and obeying commands. There was no sign of external compression of the eyes during and after surgery.

**Figure 2.**
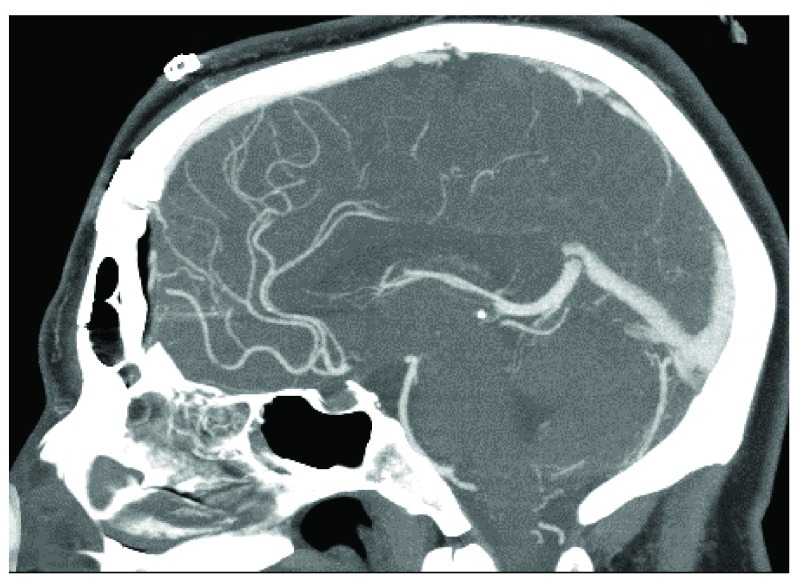
Postoperative computerized tomographic angiography showing normal perfusion of both ophthalmic artery and vein, no hemorrhage or signs of cerebral ischemia or edema, just evidence of AVM resection.

On awakening in the recovery room, the patient complained of blindness in her right eye. Immediate CT angiogram of the brain was inconclusive showing normal perfusion of both ophthalmic artery and vein, no hemorrhage or signs of cerebral ischemia or edema, just evidence of AVM resection ( Figure 2). Ophthalmology was immediately consulted. Examination of the right eye showed a posterior ION in association with a relative afferent pupillary defect (RAPD), but fundoscopy was normal without optic disc edema or cherry-red spot. Upon diagnosis of ION, the patient immediately received digital massage of the right eye once for a few minutes. Anti-glaucoma treatment (timolol maleate ophthalmic solution 0.5% applied to the affected eye twice daily for 2 days) was given to decrease intraocular pressure (IOP). Mild postoperative pain was treated with small doses of intravenous fentanyl; otherwise, the patient did not have any postoperative complications. On the following day, her vision had somewhat improved; however, the right temporal visual field was still missing. Examination of the retina was again normal, and no further treatment was administered. She was discharged from the hospital 2 days after surgery. In a follow-up visit after 3 months, she was neurologically intact, and her vision was better. Her visual field had returned to normal with just some blurring of vision in the right eye. To our knowledge, no formal assessment of visual fields was performed after discharge.

## Discussion

This patient experienced acute unilateral loss of visual acuity and visual field after AVM surgery in the supine position. Findings were consistent with the diagnosis of POVL due to posterior ION with RAPD. Fundo- and retinoscopy of the affected eye were normal, and there was no sign of external pressure on the ocular globe. Our patient did not have risk factors for POVL, except for mild obesity. Vision loss did not seem to be related to the actual AVM or its surgical removal. Given the characteristics and location of the AVM and the surgical technique used in this case, it seems unlikely that an intraoperative insult to the optic nerve or its blood supply via direct trauma to the nerve or compromise of the blood supply with edema or brain retractors was a factor in the development of POVL. In a review of the literature, we only found one case of unilateral visual loss after intracranial aneurysm surgery (ASA POVL Registry) ^4^, suggesting that POVL is a very rare complication of intracranial neurovascular surgery in the supine position. Due to the multitude of perioperative and patient variables, it is difficult to definitively ascertain the etiology of POVL in this case.

**Figure 3.**
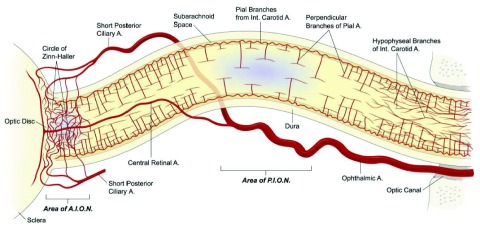
Diagram of the orbital optic nerve with its vascular supply. The areas involved in the ischemic insults are highlighted in purple. The anterior optic nerve is supplied by multiple sources while only the small pial branches supply the mid-orbital optic nerve. It is the mid-orbital region of the optic nerve that is involved in PION. The pial branches are found with variable density and in an unusual perpendicular T-shaped pattern, characteristic of a low pressure system. There is low density of arteriolar and capillary supply to this mid-orbital segment compared with the canalicular or retro-bulbar segments of the optic nerve. A., Artery; AION, Anterior Ischemic Optic Neuropathy; PION, Posterior Ischemic Optic Neuropathy. Image courtesy of Baig M.N. *et al.*, 2007, Vision loss after spine surgery: review of the literature and recommendations, *Neurosurgical Focus*, November, 23 (5): E15 ^6^. Re-print permission has been obtained from the Journal of Neurosurgery Publishing Group. This figure is not subject to the CC license that the rest of this paper is subject to. Any use of this figure is subject to obtaining the necessary permissions from the Journal of Neurosurgery Publishing Group.

Figure 3 shows a diagram of the blood supply of the optic nerve; if any impairment of blood supply occurs, ION may develop. POVL may present as both uni- and bilateral. An association with systemic diseases, such as hypertension, diabetes, hypercholesterolemia, or atherosclerosis, is not well documented. Several potential causes of POVL have been described. Intraoperative corneal trauma may result in irritation, abrasion, or even laceration of the eye. Preventive measures include taping the eyes shut and careful patient positioning. Intraoperative stroke involving the visual tracts or the visual cortex may lead to hemianopsia and cortical blindness. Cerebral ischemia may be due to prolonged systemic hypotension or thromboembolism. Arteriosclerosis-related embolism also plays an important role in the etiology of central retinal artery occlusion (CRAO) ^6^; however, CRAO may be caused by an acute and severe rise of IOP found in trauma or direct external pressure to the ocular globe as well. Clinical findings in CRAO are the unilateral painless loss of vision with signs of external periorbital swelling or ecchymosis, and a pathognomic cherry-red spot at the macula. Recently, posterior reversible encephalopathy syndrome (PRES) has also been proposed as potential cause of POVL ^7^.

Risk factors that have been implicated in the development of ION in spine surgery include male sex, obesity, prolonged intraoperative hypotension, long duration of surgery, substantial intraoperative blood loss, and the excessive use of intravenous replacement fluids, or anemia ^8, 9^. The management of POVL has been described by an ASA task force ^3^.

The majority of POVL cases are reported after lumbar spine surgery ^1, 2, 6– 10^, suggesting a greater incidence of POVL associated with prone positioning. Increases in IOP during surgery in the supine position were thought to be an important factor for the development of visual loss; the current recommendation of the ASA task force ^3^ is to keep the head elevated higher than the heart and in neutral position whenever possible. Intraoperative blood loss and prolonged arterial hypotension should be avoided. Intravenous administration of colloid solutions and avoidance of excessive crystalloid infusion has been recommended. Excess crystalloid may cause tissue edema, and compromise tissue oxygenation in the orbital cone. If prolonged duration of surgery in the prone position is necessary, the eyes should be examined for external compression or swelling at regular intervals.

## Conclusion

POVL involving ION remains a rare, but devastating condition. Unfortunately, our limited knowledge of the pathophysiology restricts the treatment options. Type of surgery, patient-related and intraoperative risk factors have been identified, but were absent in this case, apart from mild obesity. Clinicians should be aware of high-risk cases for POVL such as major spine surgery in the prone position and cardiac surgery. Intraoperative head elevation, use of colloids, avoidance of excessive crystalloid infusion and prolonged arterial hypotension, correction of anemia, and staging of surgery to reduce operating time are preventive measures recommended by the ASA task force on POVL ^3^. Cases of permanent loss of vision have occurred, therefore early diagnosis and treatment are paramount to increase chances of visual recovery in the event of POVL.

## Patient consent

Written informed consent for publication of clinical details was obtained from the patient.
