# Supplementary material for: Postoperative visual loss following cerebral arteriovenous malformation surgery: a case report
Source: F1000Res. 2014 Mar 14;3:27. Originally published 2014 Jan 27. [Version 2] doi: 10.12688/f1000research.3-27.v2 (PMC3945764; doi:10.12688/f1000research.3-27.v2)
Supplement: Peer review history

# Peer review: Referee response for version 1

**Published:** 2014-02-05

**Authors:** Sergio Bergese

**Affiliations:** 1 Department of Anesthesiology, Ohio State University, Columbus, OH, USA

After reading " **Postoperative visual loss following cerebral arteriovenous malformation surgery: a case report**" I have some minor additions to propose to the authors:

1. Extend discussions based on patient medical history linked to POVL  - neurological, ophthalmological, and concomitant medication.
   
2. Explain the standardized grading scheme: Spetzler-Martin.

The authors covers a topic of significant scientific interest, with original data and good quality of presentation.

I have read this submission. I believe that I have an appropriate level of expertise to confirm that it is of an acceptable scientific standard.

# Peer review: Referee response for version 1

**Published:** 2014-02-05

**Authors:** Lorri A Lee

**Affiliations:** 1 Department of Anesthesiology and Pain Medicine, University of Washington, Seattle, WA, USA

This case report of PION occurring ipsilaterally after a resection of a frontal AVM is very interesting. It is a reminder that PION can occur in many different types of procedures outside spine, cardiac, and head and neck surgery. During craniotomies, more direct insult to the blood supply of the optic nerve may be an additional etiologic factor via direct trauma to the nerve or blood supply, or compromise of the blood supply with edema or brain retractors. It is difficult to ascertain with the available information the proximity of the AVM to the optic nerve and its blood supply.

For the readers of this case report, it might be helpful to discuss the blood supply of the optic nerve with a diagram, including both the superior hypophyseal artery and the opthalmic artery and branches. The patient's preoperative angiogram and MRI/CT of the head, showing the proximity of the AVM and venous drainage to the optic nerve and its blood supply would be helpful information for the clinician.

Other useful information would include a statement re: any other postoperative complications; any signs of edema/ischemia on later CT/MRI of the head; later opthalmologic findings such as optic nerve pallor on fundoscopy (confirmation that it was PION); and if formal assessment of visual fields was performed after discharge.

It will remain difficult to definitively ascertain the etiology of PION in this case, even if all the requested information is provided. Fortunately, perioperative PION is relatively rare; however, its low incidence, high severity of injury, and the multitude of perioperative and patient variables make it exceedingly unlikely that studies with a high level of evidence will be conducted in the future.  Animal models of PION simulating perioperative conditions may provide important insights into etiology, prevention and treatment of this problem. Unlike most patients with perioperative PION, this patient seems to have had good recovery of vision.

(N.B. The lack of reports of craniotomies and POVL in the ASA POVL Registry is more likely a result of exclusion of cases in that Registry where direct trauma to the optic nerve or its blood supply was possible.)

I have read this submission. I believe that I have an appropriate level of expertise to confirm that it is of an acceptable scientific standard.

# Peer review: Referee response for version 1

**Published:** 2014-01-29

**Authors:** John Gaudet Van Driest

**Affiliations:** 1 Division of Neurosurgical Anesthesia, Columbia University Medical Center, New York, NY, USA

The authors report a case of partially reversible right unilateral visual loss following elective resection of a right frontal arteriovenous malformation. Immediate ophthalmologic assessment in recovery is compatible with ischemic optic neuropathy (ION), a condition not commonly reported following neurosurgical surgery in supine position. The pathophysiology of ION remains unclear; it is most likely multifactorial.

 

**Abstract**

Although clearly specified in the case description, I would clarify the abstract by adding a couple of details about the procedure: right frontal craniotomy for resection of an AVM in supine position, under general anesthesia. This is just too properly set the table before digging in, so to speak… Also, I would mention that the patient partially recovered vision 3 months after surgery, for the sake of completeness.

 

**Introduction**

I would leave the specifics of ION (presentation, assessment, causes & mechanisms) for the discussion altogether. POVL prevalence reports (0.0012% general surgery; 0.03% spine surgery; 0.086% cardiac surgery) and general classes of POVL (corneal, retinal including CRAO, optic, chiasma and tracts, cortical) are all that is needed for the introduction here.

 

**Case description**

Since obesity is a known risk factor for ION, I would mention the patient’s height & weight. Were there any visual issues at baseline? I assume there weren’t any, but I would mention it anyway. Were any of the AVM feeders embolized prior to craniotomy? Were there any other abnormalities noticed on the cerebral angiogram? In particular, since the AVM was in proximity to the right optic area; were there any arterial branches coming off the feeders? If so, could arterial exclusion have caused optic ischemia? Was the circle of Willis normal on preoperative control? We typically perform an immediate postoperative angiogram following AVM resection to confirm complete resection of the AVM, as well as to assess perfusion of normal vessels. Did you perform such an exam before emergence and extubation? If so, I would mention it with a brief description of results.

 

The discussion about potential mechanisms of ION is interesting. It appears to me that ION is a multifactorial process combining the focal effects of arterial hypotension, venous hypertension, and decreased oxygen capacity. Arterial blood pressure and oxygen capacity seem to have been managed properly in this case. The combination of elevated intra-ocular pressures and borderline low arterial pressures puts the optic nerve at risk of ischemia. You mention anti-glaucoma therapy having potentially helped to improve perfusion of the optic nerve; was this patient known for glaucoma? Venous hypertension remains a possibility; how was the head positioned during the case? Did mechanical ventilation require high peak pressures restricting cerebral venous drainage?

 

In any case, I believe this case report deserves an ‘Approved’ status but needs minor additions imposed by the specific location of the AVM relative to the visual loss. Mainly:

1. minor details about patient at baseline (height & weight, visual status)

2. preoperative angiographic assessment (feeders, Willis) and management (embolization)

3. postoperative angiographic assessment, if any.

The general discussion about ION is interesting and most relevant to this case. I found it clear and well referenced.

I have read this submission. I believe that I have an appropriate level of expertise to confirm that it is of an acceptable scientific standard.
